# Abundance and Species Diversity of Fungi in Rivers with Various Contaminations

**DOI:** 10.1007/s00284-017-1427-3

**Published:** 2017-12-30

**Authors:** A. Pietryczuk, A. Cudowski, T. Hauschild, M. Świsłocka, A. Więcko, M. Karpowicz

**Affiliations:** 1 Department of Hydrobiology, Institute of Biology, University of Białystok, Ciołkowskiego 1J, 15-245 Białystok, Poland; 2 Department of Microbiology, Institute of Biology, University of Białystok, Ciołkowskiego 1J, 15-245 Białystok, Poland; 3 Department of Molecular Zoology, Institute of Biology, University of Białystok, Ciołkowskiego 1J, 15-245 Białystok, Poland

## Abstract

The main objective of this work was to determine the abundance and species diversity of fungi in the waters of selected rivers of Central Europe, NE Poland (Augustów Lakeland), differing in size, physical and chemical properties, and streamflow rate. The minimum abundance of fungi in the analysed rivers was recorded for a river with low concentration of organic matter (8200 CFU/mL, Czarna Hańcza River), and maximum for a strongly anthropogenically polluted river (24,800 CFU/mL, Kamienny Bród River). A total of 49 fungal species were identified based on PCR ITS-RFLP and DNA sequencing methods. However, RFLP-PCR method has proved to be sufficient to determine the species of 34 fungi. The highest taxonomic diversity was determined for the waters abundant in organic matter (Piecówka and Rospuda Rivers), and the lowest for rivers poor in organic matter (Netta and Czarna Hańcza Rivers). From the 49 identified species, 47% were aquatic hyphomycetes, and 18% were potentially pathogenic fungi mainly occurring in the waters of polluted rivers with increased organic matter concentrations. Moreover, a higher number of fungal taxa were recorded in fluvial waters distinguished by higher streamflow rate, and therefore, stronger water turbulence. The study results suggest that the most important factors influencing the structure of mycoplankton in rivers include pH of water, content of organic matter, degree of anthropogenic pollution, and streamflow rate.

## Introduction

Fungi fulfil very substantial functions in such environments. In most of all they are important elements of the microbial loop. Together with bacteria, they initiate the decomposition of organic matter, particularly that of plant origin, preparing the substratum to be inhabited by other organisms, particularly invertebrates [41]. It has been evidenced that only some few hours after falling into streams, leaves are covered with a biofilm of microorganisms with a definite prevalence of fungi [30]. Moreover, fungi inhabiting water ecosystems actively participate in the production of autochthonic humic substances [11]. These microorganisms also actively participate in the circulation of nutrients such as nitrogen and phosphorus [8]. They can be suspended in the water depths, settled on the bottom or periphyton, be directly supplied to waters with surface runoff, or be of anthropogenic origin. Certain fungal species supplied to the water depths cause diseases in fish and other aquatic animals, as well as in humans [18]. On the other hand, they have been evidenced to actively participate in the biotransformation of xenobiotics [24] and heavy metals [41] supplied to the aquatic environment, potentially contributing to the alleviation of the effects of anthropogenic stress, and improving water quality. Due to this, a lot of authors have proposed to include these organisms in the group of bioindicators of anthropogenic alterations in the monitoring of the ecological state of aquatic ecosystems [5, 32, 39], and the sanitary state of waters [4, 10]. Research regarding the abundance [21, 33] and taxonomic identification of aquatic fungi in various types of waters, and particularly in lakes with varied trophic status [6, 20, 34], has been increasingly frequent in the recent years. Attempts to explain the effect of physical and chemical water parameters on the species diversity and abundance of mycoplankton, particularly in flowing waters, are very scarce. That is why the main goal of this manuscript is identification of taxonomic structure and abundance of fungi in rivers with various streamflow rate and contaminations determined by nutrients (C, N and P) and sulphate and chloride ions which are indicators of anthropogenic pollution. It will be helpful to understanding of functioning aquatic fungi in different type of flowing surface water. It could allow for the future application of this group of organisms as bioindicators of the ecological state, and indicators of the sanitary quality of waters.

## Materials and Methods

### Study Area

The study area included a group of five rivers located in Central Europe (Poland). The Rospuda, Netta, and Kamienny Bród Rivers are located in the catchment of the Vistula River, and the Piecówka and Czarna Hańcza Rivers in the catchment of the Neman River. The Kamienny Bród River flows through areas under agricultural use, and the Piecówka and Rospuda Rivers through developed areas supplying household sewage to the rivers. The Czarna Hańcza and Netta Rivers flow through areas without human intervention. The monitoring of the rivers was conducted in the hydrological year 2013 in favourable meteorological conditions permitting credible results.

### Collection of Material for Analysis

Al water samples for chemical and molecular analysis were collected four times in different hydrological seasons in January, May, August, and October of the hydrological year 2013 using a Limnos sampler from a depth of 0.5 m in the midstream. The samples were collected from each river in two places (the Rospuda River—N53°98 E22°80 and N53°90 E22°95, the Netta River—N53°80 E22°97 and N53°67 E22°89, the Kamienny Bród River—N53°93 E22°82 and N53°87 E22°90, the Piecówka River—N53°88 E23°45 and N53°85 E23°42, the Czarna Hańcza River—N53°89 E23°41 and N54°24 E22°80). Moreover, the streamflow rate (SQ) of river waters was measured using an ADC digital current meter (OTT) (Table 1). The water samples for mycological and chemical analyzes were transported to the laboratory in glass bottles (1 L) in a cool (0–4 °C) and in the dark with accordance to the PN-EN ISO 19458, ISO 5667-5: 2003.

**Table 1:** The average values (in bold) and minimum–maximum of streamflow quantity (SQ) of analysed rivers water

Rivers	Kamienny Bród	Piecówka	Rospuda	Netta	Czarna Hańcza
SQ (m^3^/s)	**0.63**	**0.54**	**8.09**	**8.13**	**7.30**
	0.45–0.84	0.46–0.63	7.79–8.61	7.18–9.19	6.62–7.59

### The Physico-chemical Water Quality

Water temperature, electrolytic conductivity (EC), oxygen saturation, dissolved oxygen concentration and pH were measured in the field using a HQ40D Hach Lange meter. Analysis of the basic chemical water parameters was conducted in laboratory. They determined concentrations of sulphate(VI), chloride and phosphorous ions as well as nitrate(V), nitrate(III), ammonium which allowed for calculating the total inorganic nitrogen (TIN) according to standards methods [1]. Chlorophyll *a* concentration was determined according to PN-86/C-05560/02 [35]. The concentration of dissolved organic carbon (DOC) was determined by the high-temperature catalytic method of incineration in a TOC-5050A analyser (Shimadzu), and particulate organic carbon (POC) was determined by the chromate method [7]. Phenols concentration was determined according to Lowry et al. [26] method.

### The Abundance of Fungi

To estimate fungal abundance, 250 µL of unfiltered water, diluted to ratios of 1:10 and 1:100, were placed on Sabouraud agar plates enriched in chloramphenicol (0.05 g/L) and incubated for 5 days at either 37, 25 or 5 °C (due to differences in optimum growth temperature). After incubation, the numbers of colonies (CFU/mL) were determined [12].

### The Taxonomic Structure of Fungi

DNA was isolated from fungal cells using Genomic Mini AX Yeast and Bead-Beat Micro Gravity DNA Isolation Kit (A&A Biotechnology) according to the manufacturer’s protocol. The reaction mixture for PCR amplification per one sample contained 2 μL of isolated DNA, 10 pmol of ITS1 primer (5′-TCCGTAGGTGAACCTGCGG-3′), 10 pmol of ITS4 primer (5′-TCCTCCGCTTATTGATATGC-3′) [17], 11.75 μL of nuclease-free water (A&A Biotechnology), and 12.5 μL of PCR Master MixPlus (A&A Biotechnology). The PCR reaction was performed in a GeneAmp PCR System 9600 thermal cycler (Applied Biosystems) under the following conditions: initial denaturation for 3 min at 95 °C, 40 cycles consisting of denaturation at 95 °C for 1 min, annealing at 52 °C for 1 min, extension at 72 °C for 1 min and final extension step at 72 °C for 10 min. The presence of a PCR product was confirmed by 1% (wt/vol) agarose gel electrophoresis and visualised with ethidium bromide (Gel DOC XR(TM) Imaging System, Bio-Rad). Amplified PCR products were sequenced with the BigDye™ Terminator Cycle Sequencing Ready Reaction Kit v 3.1 (Applied Biosystems) in both the forward and reverse directions using the amplification primers. The sequencing reaction proceeded with the following sequencing profile parameters: preliminary denaturation at temperature 94 °C for 3 min followed by 25 cycles of denaturation at 94 °C for 30 s, primer attachment at 50 °C for 15 s and elongation at 60 °C for 4 min. Unincorporated dideoxynucleotides were eliminated from the sequencing reaction using the ExTerminator Kit (A&A Biotechnology) and then the sequencing products were subjected to electrophoresis and direct analysis in an automatic sequencer (Applied Biosystems). To read, adjust and align the sequence, commonly used computer software was used: Chromas Lite v.2.01 (Technelysium Pty Ltd, 2005) and BioEdit Sequence Alignment Editor v.7.0.1 [19].

The DNA digestions were performed with 10 µL of PCR product in a total volume of 15 µL with 1× reaction buffer and 10 U of *Eco*RI endonuclease (Sigma-Aldrich) for 2 h at 37 °C. The resulting fragments were separated by electrophoresis on a 2% agarose gel and visualised under UV light after ethidium bromide staining [14]. The lengths of the products were evaluated using a 1000 pb DNA ladder. The results were archived using Gel Doc 2000 equipment (Bio-Rad) and the software program Quantity One. The lengths of the DNA fragments separated on a gel after digestion were compared to the fragment lengths of the reference strains in GenBank.

### Statistical Analysis

To investigate the relationships between environmental and biological data, a redundancy analysis (RDA) was carried out. RDA is an enhancement of the commonly applied principal component analysis (PCA); however, unlike PCA, RDA allows direct analysis of biotic-environmental components [42, 43]. To test whether RDA analysis was appropriate for the dataset, the data were first tested for normality (Kolmogorov–Smirnov test). DCA was used first to determine the pattern of variability in the studied assemblages: if the length of the first gradient was > 2 standard deviations, we assumed a unimodal variation; a length < 2 SD indicates a linear variation [25]. The length of the first gradient for the fungi communities was 1.71 SD, indicating linear variation, justifying RDA. Correlation coefficients were calculated with Pearson ranks. The analysis of existing differences among rivers involved multi-dimensional analysis, specifically, cluster analysis, where the Euclidean distance was adopted as the probability measure and the Ward’s method as the clustering procedure. Statistical calculations were performed using Statistica version 7 and CANOCO for Windows 4.5 software.

## Results

**Fig. 1:**
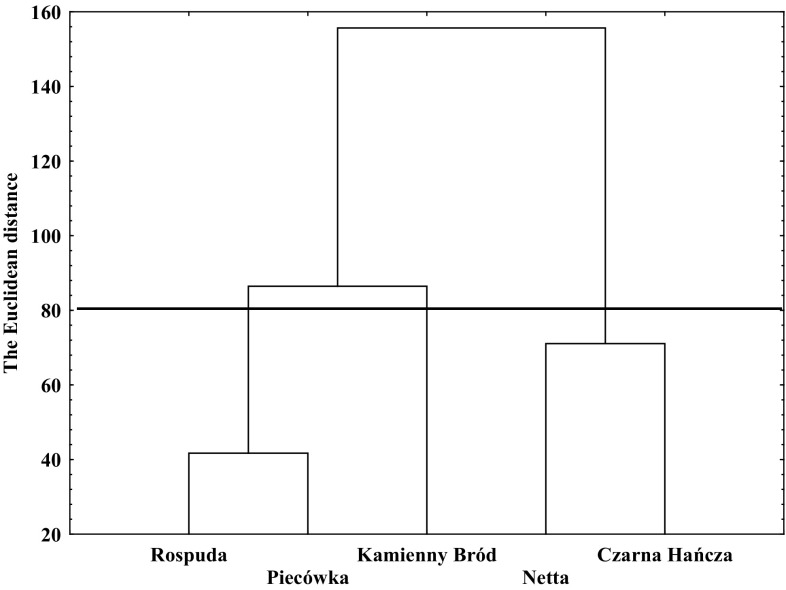
Result of the cluster analysis of the the selected rivers of the Augustów Laceland in NE Poland

Based on the physico-chemical analysis of river waters, three types of habitat have been distinguished which were characterized by different water quality (Fig. 1). The Rospuda and Piecówka Rivers were the first group, which was characterized by increased organic matter content (Table 1). The second group included the Kamienny Bród River, which was characterized by high concentrations of TIN, chloride and sulphate(VI) ions as well as EC (Table 1). The last group was represented by Czarna Hańcza and Netta Rivers, which have low concentrations of carbon, nitrogen, phosphorous and sulphate(VI) and chloride ions (Table 1).

A total of 49 fungal species were identified based on PCR ITS–RFLP and DNA sequencing methods. However, RFLP–PCR method has proved to be sufficient to determine the species of 34 fungi. The highest taxonomic diversity was determined for the waters of the Piecówka and Rospuda Rivers, where 32 and 37 species were recorded, respectively. The dominant species in both rivers were *Helicoon gigantisporum, Heliscus lugdunensis*, and *Tetracladium maxilliforme*. Moreover, *Achlya* sp. preponderated in Piecówka River and *Pythium* sp. in Rospuda River. The lowest diversity was found in the Netta and Czarna Hańcza Rivers (20 and 15 species, respectively) (Table 2). The dominant species in both rivers were *Leptomitus lacteus* and *Lemonniera aquatica*. It was found that *Clavariopsis aquatica* was the most common species in Kamienny Bród River, where 22 species were identified. From the 49 identified species, 23 (47%) matched with species belonging to the aquatic hyphomycetes group. Moreover, the studied rivers also showed occurrence of potentially pathogenic fungi, dominated by *Candida albicans, Acremonium implicatum, Chrysosporium keratinophilum, Trichosporon cutaneum*, and *Rhodothorula rubra*, particularly abundant in polluted rivers (Table 2). The presence of fungal species belonging to the aquatic hyphomycetes group was recorded in the waters of practically all of the studied rivers.

**Table 2:** Taxonomic diversity of aquatic fungi in the selected rivers of the Augustów Lakeland (accession numbers of the reference strains in GenBank are provided in brackets) italic rows—potentially pathogenic fungi

No	Species	Rivers				
		Kamienny Bród	Piecówka	Rospuda	Netta	Czarna Hańcza
1	*Achlya americana* (HQ643084.1)	×	×	×		
2	*Achlya flagellata* (HQ643097.1)		×	×		
3	*Achlya oligacantha* (HQ643101.1)		×	×	×	
4	*Acremonium implicatum* *(JQ910163.1)*	*×*		*×*		
5	*Alatospora acuminata* (KF730797.1)			×	×	
6	*Alatospora flagellata* (KC834041.1)			×	×	
7	*Alternaria alternata* (AY354228.1)	×	×	×		×
8	*Anguillospora crassa* (AY148106.1)		×	×	×	×
9	*Anguillospora filiformis* (JX089472.1)			×	×	×
10	*Aphanomyces laevis* (HQ643122.1)	×	×	×		
11	*Apodachlya brachynema* (HQ643125.1)	×	×	×		
12	*Arthroderma insingulare* *(AJ877213.1)*	*×*		*×*	*×*	
13	*Aspergillus fumigatus* (FJ867935.1)		×	×	×	
14	*Aspergillus niger* (JQ929761.1)	×	×	×	×	×
15	*Candida albicans* *(EF192231.1)*	*×*	*×*	*×*		
16	*Chrysosporium keratinophilum G* *(KC923425.1)*	*×*	*×*			
17	*Clavariopsis aquatica* (GQ152143.1)	×			×	
18	*Clavatospora longibrachiata* (KF730809.1)	×			×	×
19	*Exophiala dermatitidis* *(KF996500.1)*	*×*				
20	*Flagellospora curvula* (KC834050.1)			×		×
21	*Helicoon gigantisporum* (AY916467.1)		×	×		
22	*Heliscus lugdunensis* (HQ897796.1)		×	×		
23	*Lemonniera aquatica* (KF730823.1)	×			×	×
24	*Lemoniera centrosphaera* (KC834063.1)			×	×	
25	*Leptomitus lacteus* (AF119597.1)	×				
26	*Lunulospora curvula* (JX089535.1)		×	×	×	
27	*Microsporum gypseum* *(EU151494.1)*	*×*		*×*		
28	*Penicillium funiculosum* (HQ637359.1)	×	×	×	×	
29	*Pythium aquatile* (HQ643445.1)		×	×		
30	*Pythium afertile* (HQ643416.1)	×	×			
31	*Pythium debaryanum* (HQ643519.1)	×	×	×		
32	*Pythium rostratum* (HQ643767.1)		×	×		
34	*Pythium undulatum* (AY436638.1)			×		
35	*Rhodotorula rubra* *(AB916512.1)*	*×*	*×*			
36	*Saprolegnia parasitica* (KC992717.1)	×	×	×		
37	*Tetracladium breve* (EU883431.1)		×	×	×	×
38	*Tetracladium furcatum* (GU586842.1)		×	×	×	×
39	*Tetracladium marchalianum* (EU883423.1)		×	×	×	
40	*Tetracladium maxilliforme* (KC989085.1)		×	×		
41	*Trichophyton violaceum* *(JQ322678.1)*		*×*			
42	*Trichosporon mucoides* *(FJ515218.1)*	*×*	*×*			*×*
43	*Tricladium angulatum* (AY204610.1)		×	×	×	×
44	*Tricladium patulum* (FJ000403.1)		×	×	×	×
45	*Tricladium splendens* (DQ202511.1)	×	×	×	×	
46	*Tumularia aquatica* (FJ000399.1)				×	
47	*Varicosporium delictum* (JQ412864.1)		×	×	×	×
48	*Varicosporium elodeae* (JX981463.1)			×	×	
49	*Volucrispora graminea* (AJ748690.1)		×	×	×	×

**Fig. 2:**
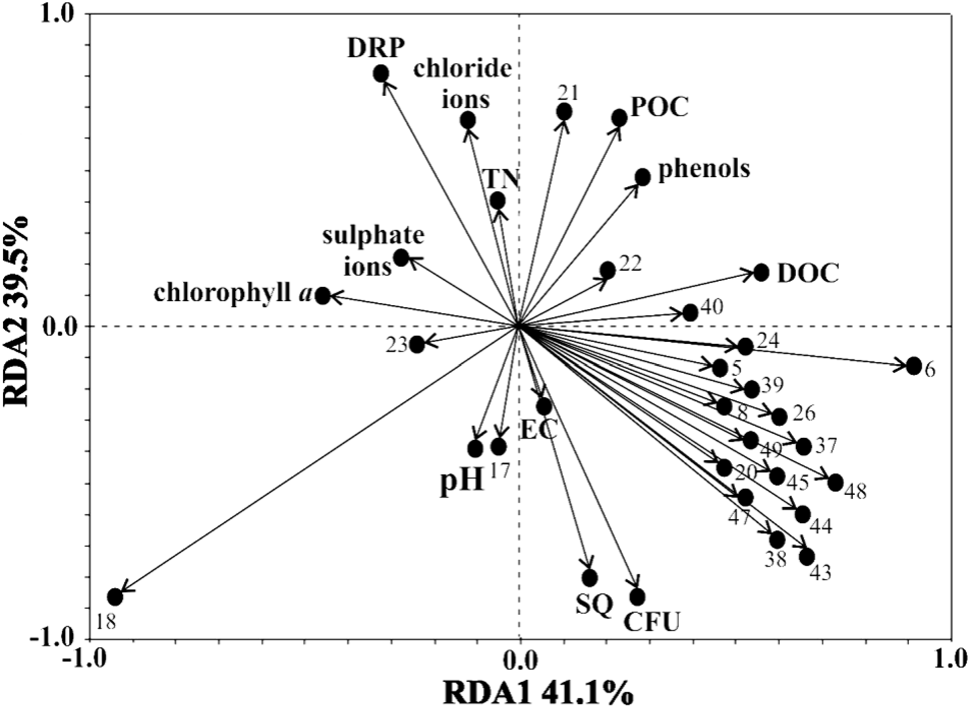
RDA of abundance (CFU) and species diversity (numbers correspond to those of a given species in Table 2) of hyphomycetes, selected physico-chemical parameters of water quality and streamflow quantity (SQ) of analysed rivers water

The total abundance of fungi in the waters of the studied rivers varied from 8200 CFU/mL in the Czarna Hańcza River to 22,800 CFU/mL in the Piecówka River (Table 3). These were positively correlated with water EC (r = 0.88, *P* < 0.01), and negatively correlated with chlorophyll *a* concentration (*r* = 0.80, *P* < 0.01), dissolved reactive phosphorus (*r* = 0.68, *P* < 0.01) and TIN (*r* = 0.83, *P* < 0.01). No statistically significant correlation was found between total fungal abundance and the concentration of DOC, POC, and phenol compounds (Fig. 2). The outcome of the RDA analysis is presented as a diplot containing environmental measurements and fungal species (Fig. 2). The first two principal components jointly accounted for 80.6% of the variance in the original variables for the waters of the analysed rivers, based on the hydrochemical parameters measured and the species composition of fungi from the aquatic hyphomycetes ecological group (Fig. 2). The RDA analysis revealed two main environmental clines (Fig. 2). Chlorophyll *a*, sulphate and chloride ions, TIN, DRP, SQ, CFU and EC formed the first cline, with chlorophyll *a*, sulphate and chloride ions, DRP, TIN vectors oriented opposite from EC, SQ and CFU. This indicates a negative correlation between these two groups of parameters. The second grouping was based on POC, DOC, phenols compounds, and pH with POC, phenols, DOC vectors oriented opposite to pH (Fig. 2).

**Table 3:** The average values (in bold) and minimum–maximum of selected parameters of rivers water quality

Parameters	Rivers				
	Kamienny Bród	Piecówka	Rospuda	Netta	Czarna Hańcza
Fungi abundance (CFU/mL)	**21,975**	**22,250**	**19,000**	**11,450**	**9175**
	18,800–24,800	19,800–24,200	17,200–20,500	10,000–12,200	8200–10,200
Chlorophyll *a* (µg/dm^3^)	**7.48**	**1.71**	**5.33**	**3.14**	**13.8**
	3.96–10.5	1.46–2.00	4.91–6.00	2.28–4.60	11.9–17.0
EC (μS/cm)	**600**	**425**	**460**	**419**	**390**
	512–679	393–460	416–499	382–500	311–462
pH	**7.74**	**7.58**	**7.57**	**7.81**	**7.85**
	7.55–8.05	7.20–7.88	7.13–8.17	7.53–8.01	7.29–8.16
POC (mgC/dm^3^)	**1.92**	**3.95**	**3.49**	**0.69**	**0.72**
	1.55–2.30	3.52–4.30	3.11–4.30	0.56–0.78	0.26–0.98
DOC (mgC/dm^3^)	**10.9**	**19.4**	**15.9**	**7.32**	**6.02**
	8.95–13.3	16.8–23.2	12.6–18.0	4.96–9.27	2.17–8.04
Phenols (mgC/dm^3^)	**0.96**	**1.11**	**0.75**	**0.58**	**0.48**
	0.85–1.06	0.95–1.30	0.55–0.89	0.48–0.68	0.37–0.59
TIN (μgN/dm^3^)	**1101**	**732**	**670**	**446**	**313**
	974–1233	616–843	586–770	357–508	234–433
DRP (μgP/dm^3^)	**133**	**123**	**118**	**96.3**	**93.9**
	106–137	92.6–134	116–121	85.3–106	90.4–95.7
Sulphate ions (mg/dm^3^)	**77.1**	**44.2**	**46.8**	**36.1**	**31.8**
	73.4–84.9	33.7–51.6	39.2–61.3	29.7–41.3	27.8–35.7
Chloride ions (mg/dm^3^)	**23.4**	**14.2**	**13.9**	**11.3**	**13.4**
	22.3–25.1	12.8–15.3	11.2–15.6	10.5–13.0	12.0–15.5

## Discussion

Research concerning the species diversity and abundance of fungi in rivers depending on their hydrochemical properties has only been sporadic so far [16, 21, 32, 33, 39]. Our research showed large species diversity of fungi in rivers water in which were identified potential pathogens and hyphomycetes, as well as zoosporic fungi belonging to genera *Achlya* sp., *Aphanomyces* sp., and *Pythium* sp.. They were predominant in the Piecówka and Rospuda Rivers. The dominance of species belonging to these fungal genera in flowing waters was also recorded by other authors [27, 45]. The abundance of fungi in the studied rivers was more than twice higher than in the lakes of the Augustów Lakeland [34], or the Augustów Canal [10]. It was caused by higher streamflow rate and water turbulence intensity result in the migration of fungi from sediments to the water depths [37]. Moreover, that is why more fungal species were identified in the river showing a higher streamflow rate (Netta River), but it is not the only factor determining the species diversity and abundance of fungi in rivers water. The research carried out on the rivers of south-west France showed that *Alatospora acuminata* and *Clavariopsis aquatica* were dominated [13] whereas in the rivers of north-east Poland analysed in the scope of the present study, their occurrence was less frequent. This may be related to the fact that both of the species prefer waters with acidic pH (4–5) [13, 38] whereas the pH of the waters of the studied rivers of the Augustów Lakeland ranges from 7.52 to 7.81. The presence of *Clavariopsis aquatica* and *Alatospora acuminata* in waters with slightly alkaline pH suggests, however, that the occurrence of the species is particularly influenced by its streamflow rate. No presence of these species was also recorded in the slightly acidic waters of the dams of the Augustów Canal [10], or the lakes of the Augustów Lakeland [34], where water streamflow is hardly identifiable. Certain fungal species develop in a broad range of pH values, as confirmed by the study of Casas and Descals [9]. Potentially pathogenic fungal species predominated in waters with the highest pH values (Kamienny Bród River, Piecówka River, and Rospuda River). This is in accordance with the literature data, suggesting that alkalisation of the environment contributes to the development of potentially pathogenic fungi [10, 44].

Another important factor determining the species diversity of fungi is organic matter which constitute the basic sources of carbon for microfungi [15, 29]. However, Solé et al. [39] evidenced that species such as: *Tetracladium marchalinum, Lemoniera centrosphaera, Alatospora flagellata*, and *Alatospora accuminata* occur in waters irrespective of organic matter concentrations. Moreover, this list of fungi can be extended by such species as: *Anguillospora crassa, Flagellospora curvula, Lunulospora curvula, Tetracladium breve, Tetracladium furcatum, Tricladium angulatum, Tricladium patulum, Tricladium splendens, Varicosporium delicatum, Varicosporium elodeae*, and *Volucrispora graminea* according to our research. According to Solé et al. [39], species not tolerating high concentrations of organic carbon include: *Anguillospora longissima, Clavariopsis aquatica, Lemoniera aquatica*, and *Clavatospora longibrachiata*, what has been confirmed by our results. The highest total abundance and number of fungal species were recorded in rivers with high organic matter concentrations, which simultaneously recorded highest EC values and concentrations of sulphate(VI) and chloride ions, TIN, dissolved reactive phosphorus, constituting an indicator of water pollution of anthropogenic and agricultural origin. Sridhar et al. [40] and Pascoal et al. [32] stated that high abundance of hyphomycetes occur in waters polluted what also has been determined by our research. Krauss et al. [22, 23] found that the polluted waters of central Germany with high concentrations of sulphate(VI), nitrate(V), and orthophosphate(V) ions, recorded the presence of as many as 20 aquatic hyphomycetes species, dominated by *Heliscus lugdunensis*. This is not in accordance with the result of our own research, according to which the occurrence of *Heliscus lugdunensis* is independent of the concentration of sulphate(VI) and TIN in the waters of the studied rivers. It has been detected that this species is characteristic for the waters rich in nutrients provide conditions favourable for the development of a high number of microorganisms species. On the other hand, certain fungal species do not develop in waters with high concentrations of nitrate(V) ions [2, 24, 32, 41]. It has also been evidenced that water pollution usually leads to a decrease in the taxonomic diversity of aquatic hyphomycetes, including complete disappearance of species such as *Tetracladium breve* or *Tricladium* sp. [23, 32] what is in agreement with the results of the present research. Certain fungal species, such as *Anguillospora crassa, Lunulospora curvula, Tetracladium breve, Tetracladium furcatum, Tricladium angulatum, Tricladium patulum, Tricladium splendens, Varicosporium delicatum*, and *Volucrispora graminea*, were found to be negatively correlated with both TIN and sulphate(VI) and chloride ions. Therefore, they seem to be species preferring clean waters or waters with a moderate degree of pollution. Moreover, the polluted rivers were characterised by high number of potential pathogenic fungi, such as *Candida albicans, Arthoderma insingulare, Exophiala dermatidis, Trichophyton violaceum*, and *Rhodothorula rubra*. High nutrients concentrations in waters, and particularly dissolved reactive phosphorus, provide perfect conditions for the development of phytoplankton [10]. On the one hand, it has been evidenced that an increase in algal biomass results in a decrease in the biomass and species diversity of aquatic fungi, which may be related to a decrease in the level of biogenes, lower oxygen concentration in water, and high sensitivity of certain fungal species to cyanobacterial toxins [3, 28]. On the other hand, fungi may inhibit algal growth, and particularly that of diatoms and cyanobacteria [36]. This is suggested by the results of the present research that revealed a negative correlation between the total abundance of fungi and chlorophyll *a* concentration, constituting an indicator of algal biomass. A reversely proportional correlation was also recorded between chlorophyll *a* concentration and certain hyphomycetes species. This also seems to be confirmed by the recorded negative correlation between these fungal species and TIN, necessary for the growth of algae and cyanobacteria. The presence of certain species in waters, however, is largely independent of the concentrations of chlorophyll *a* or TIN. These include among others: *Clavariopsis aquatica, Flagellospora curvula, Lemonniera aquatica, Clavatospora longibrachiata*, and *Heliscus lugdunensis*. The species probably developed mechanisms permitting their functioning in the environment containing cyanobacterial toxins [28, 31, 39].

The study results show that the minimum fungi abundance was in a river with low concentration of organic matter, but the highest fungal taxonomic biodiversity was in the waters abundant in organic matter and in rivers distinguished by higher streamflow quantity. Potentially pathogenic fungi mainly occur in the waters of polluted rivers. Moreover, factors affecting the structure of mycoplankton include water pH, content of chlorophyll *a* concentrations. The study results presented in this paper seem to provide another important reason for including data on the abundance and species diversity of mycoplankton in the assessment of the ecological and sanitary state of surface waters, in particular species of aquatic fungi that are potential pathogens.
